# Gastrointestinal Stromal Tumor Masquerading As Acute Decompensated Heart Failure: A Challenging Diagnosis

**DOI:** 10.7759/cureus.46022

**Published:** 2023-09-26

**Authors:** Christopher Marsalisi, Samantha Isern, Loruanma Lam, Carmen Isache

**Affiliations:** 1 Internal Medicine, University of Florida College of Medicine – Jacksonville, Jacksonville, USA; 2 Infectious Diseases, University of Florida College of Medicine – Jacksonville, Jacksonville, USA

**Keywords:** mesenchymal neoplasm, adult gastroenterology, intraperitoneal mass, abdominal radiology, gastrointestinal stromal tumor (gist)

## Abstract

Gastrointestinal stromal tumors (GISTs) are rare lesions of the gastrointestinal tract that have a strong predisposition to the stomach and small intestine. We present a case of an 89-year-old female who initially presented to the emergency room with signs and symptoms of acute decompensated heart failure (HF) and was later discovered to have a 23-centimeter GIST in her abdominal cavity. This case emphasizes the implications of large intraperitoneal neoplasms and the unique constellation of symptoms they may present with.

## Introduction

Gastrointestinal stromal tumors (GISTs) are rare lesions of the gastrointestinal tract that comprise about 0.1-3% of malignancies in this anatomical region [1]. These tumors are believed to originate from the interstitial cells of Cajal which play an important role in the regulation of small intestine motility [1]. A majority of GISTs may not metastasize, but an estimated 10-30% may have metastatic lesions out of the primary site. Uncommon sites of primary GISTs, comprising about 5% of cases, include the rectum, esophagus, omentum, and mesentery.

Malignant GIST lesions commonly metastasize to organs including the liver and peritoneum and rarely to other places like the lungs within 10-15 years following primary identification [2]. GISTs are commonly discovered incidentally in radiographic studies. Symptoms vary depending on location, size, and whether the tumor is benign or malignant. Some common signs and symptoms include abdominal pain or discomfort, melena, nausea, vomiting, fatigue, unexplained weight loss, and a feeling of fullness or bloating [3]. In contrast, some GISTs may progress slowly until they have reached an advanced stage. The formal diagnosis of GISTs requires a biopsy of the lesion which usually demonstrates spindle cell histology; however, about 25% of neoplasms are morphologically epithelioid [3].

The development of GISTs is often associated with a specific gene mutation of the interstitial cells of Cajal. These cells located throughout the gastrointestinal tract are Kit and Kit-ligand dependent which is the gene responsible for the production of the receptor tyrosine kinases. A gain of function mutation in this gene is observed within about 80% of GIST cases which, in turn, results in cellular neoplasia [2]. These intricacies are of utmost importance when considering the management of GIST, especially if surgical excision (the mainstay of treatment) is not feasible. Many inoperable cases are complicated by metastasis, recurrence, or comorbid medical conditions resulting in high-risk surgery. Although a heterogeneous disease, the primary molecular targeted therapy for non-resectable GISTs is imatinib. Imatinib is a tyrosine kinase inhibitor that has consistently demonstrated a durable response in GIST over a 10-year period [2,4,5]. In cases of imatinib resistance, other therapies (e.g., radiofrequency or arterial embolization) should be considered along with second-line pharmaceutical therapies including sunitinib or regorafenib [2].

The literature detailing the prognosis of GISTs is optimistic, with a five-year survival rate estimated at up to 93%. There are many factors to consider in regard to prognostication; however, the main variables are typically the size, location, and mitotic rate of the tumor [6]. Despite the high survival rate, several circumstances complicate treatment and account for poor outcomes associated with GISTs including spontaneous rupture of the neoplasm and mass effect leading to neighboring end-organ damage. In a publication detailing the cases of 92 patients who suffered emergency complications of GISTs, 72% of patients presented with gastrointestinal bleeding from the mass, while the remainder were found to have intestinal obstruction [7].

Although many GISTs do not progress to serious life-threatening conditions, there are many instances where these tumors result in dysfunction of neighboring organs. In the presented case, we discuss the clinical course of a patient with a high-risk GIST which resulted in cardiopulmonary decompensation due to significant mass effect.

## Case presentation

An 89-year-old female with a medical history of hyperlipidemia and osteopenia presented to the hospital with a five-day history of worsening lower extremity edema along with dyspnea on exertion and orthopnea. Upon initial evaluation, the patient endorsed an unquantified amount of weight loss over several months which she attributed to early satiety and generalized abdominal pain. On exam, the patient had bilateral 2+ pitting edema extending up to her pretibial region and diffuse abdominal distention with associated tenderness.

**Figure 1:**
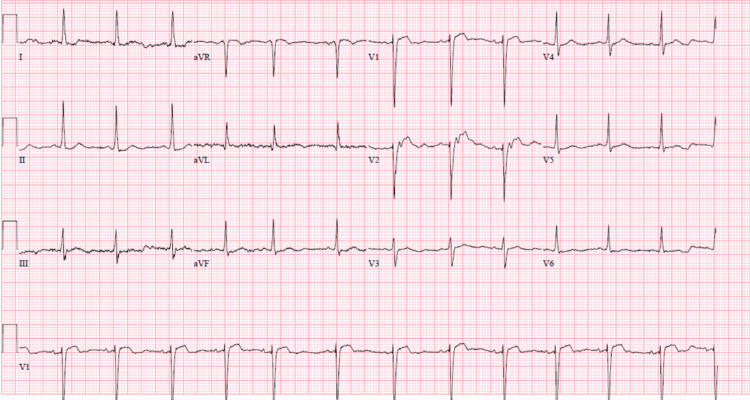
EKG demonstrating left ventricular hypertrophy EKG demonstrating left ventricular hypertrophy based on Sokolow-Lion criteria with S wave in lead V1 and R wave in lead V5 measuring 35 mm

**Figure 2:**
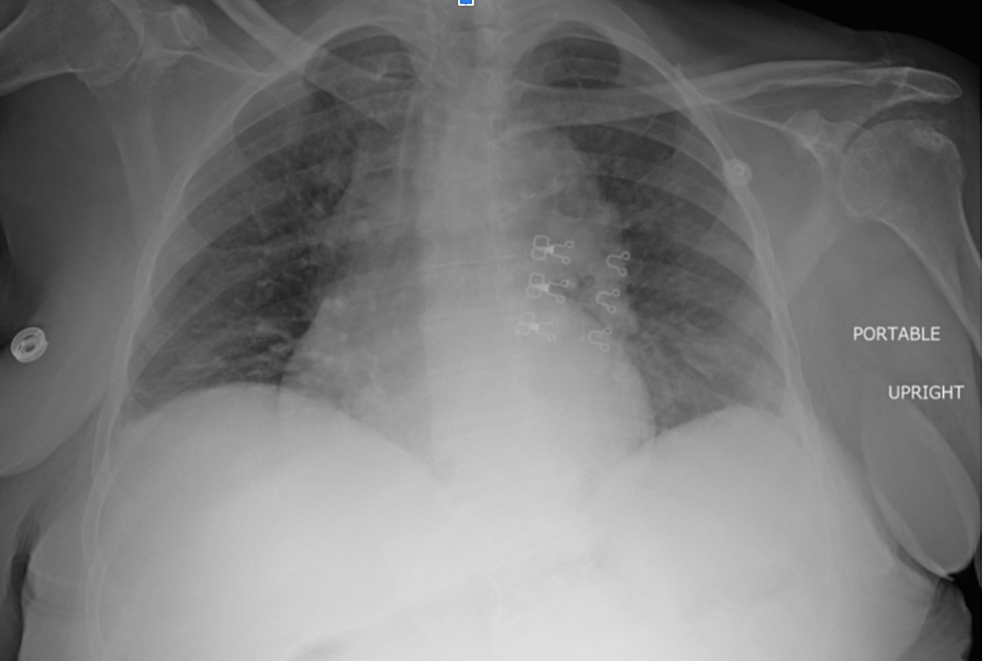
Portable chest X-ray Initial chest X-ray demonstrating diffuse interstitial lung prominence with an elevated hemidiaphragm suggesting low lung inspiration. Moreover, enlarged right atrium, enlarged peripheral hypervascularity, and pruning of the pulmonary vessels can be appreciated

Initial laboratory analysis indicated hyponatremia, hypomagnesemia, elevated NT-proBNP of 1,324 PG/mL (reference range <450 pg/mL), and iron deficiency anemia. The EKG demonstrated normal sinus rhythm with left ventricular hypertrophy (as per Sokolow-Lion criteria) (Figure 1). The initial chest X-ray was concerned for mild diffuse interstitial lung prominence likely accentuated by low lung inspiration (Figure 2). A bedside cardiac ultrasound of the cardiac chambers showed a mildly reduced ejection fraction estimated to be 40-45% based on endpoint septal separation. Evaluation of the patient’s inferior vena cava (IVC) demonstrated a large hypodense lesion neighboring the hepatic parenchyma.

**Figure 3:**
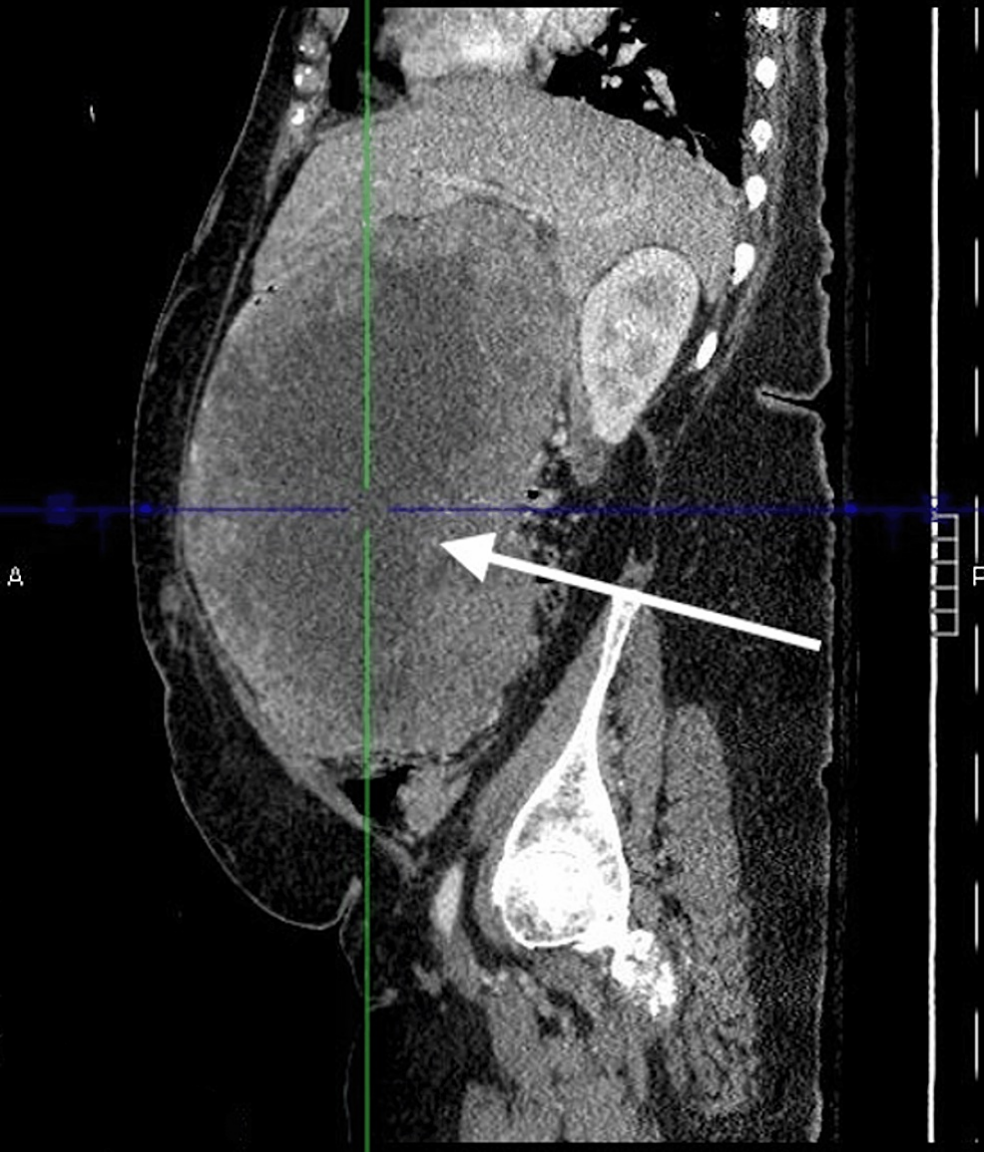
CT abdomen and pelvis CT Abdomen/pelvis sagittal plane with the white arrow pointing to a large intraperitoneal mass with central necrosis and peripheral enhancement

**Figure 4:**
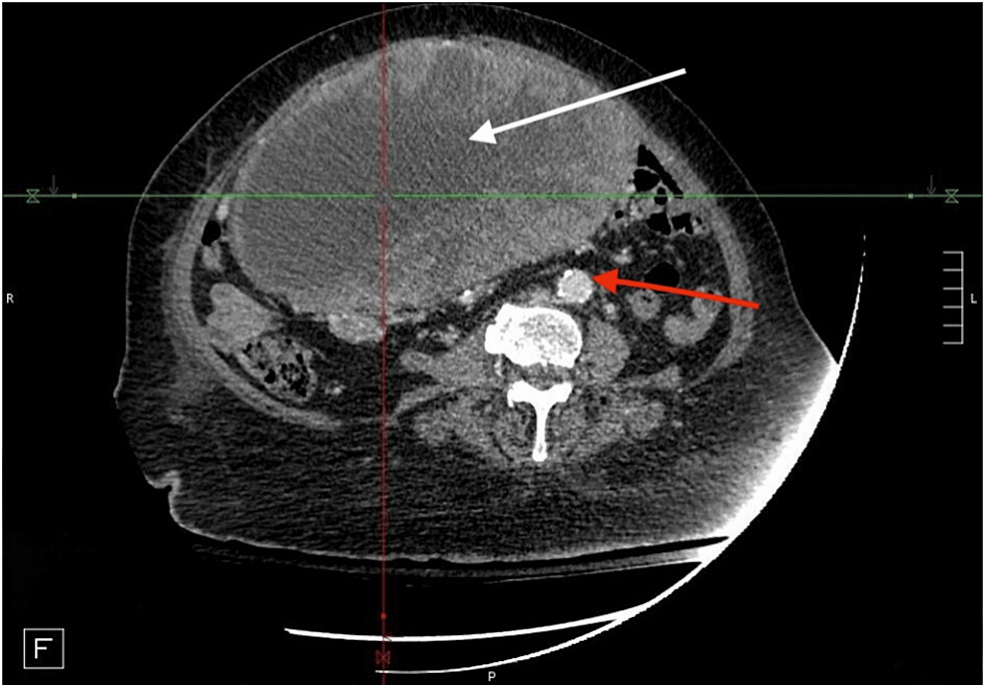
CT abdomen and pelvis CT Abdomen/pelvis horizontal/transverse plane re-demonstrating the large intraperitoneal mass (white arrow) marked with central necrosis and peripheral enhancement and lateral shift of the descending aorta from its medial baseline (red arrow)

**Figure 5:**
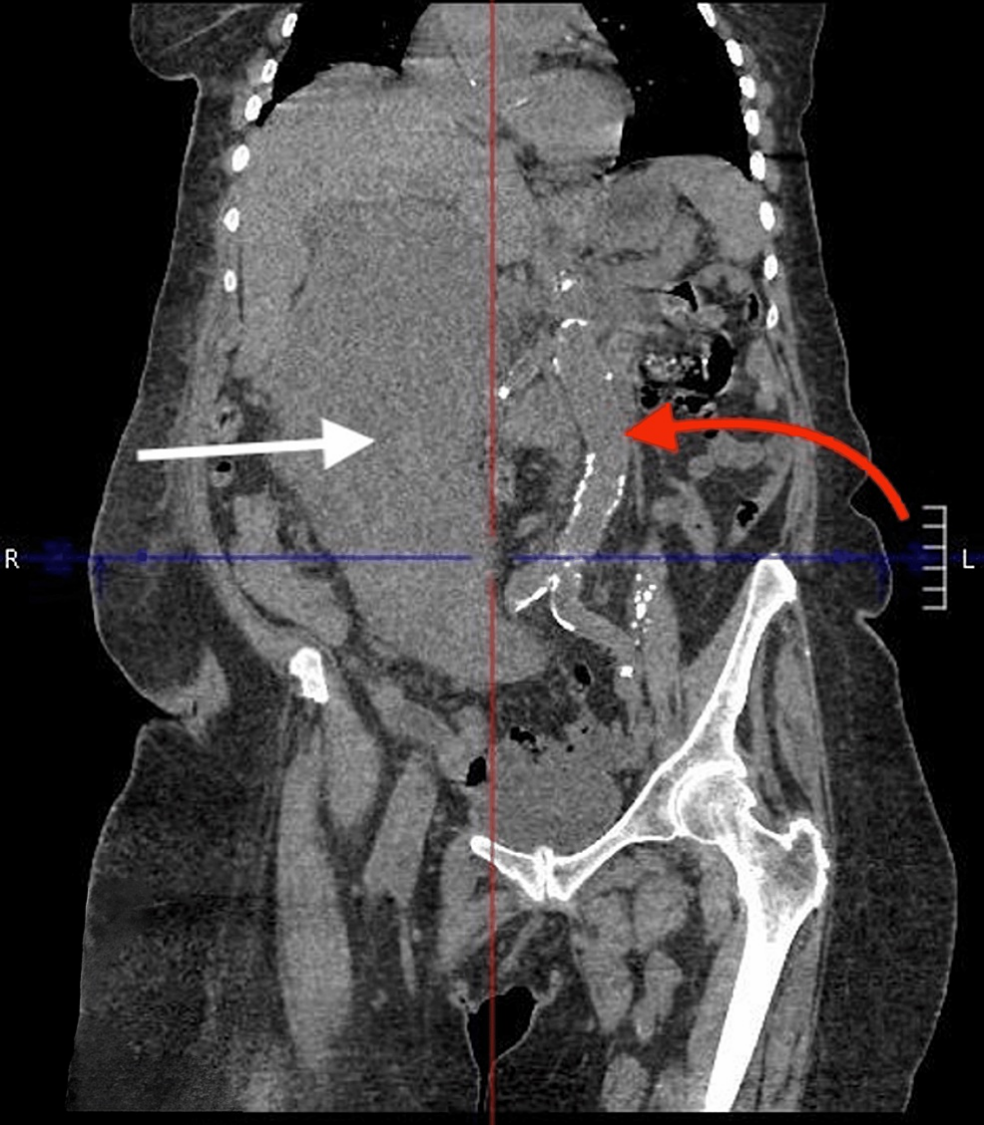
CT abdomen and pelvis CT Abdomen/pelvis coronal plane with the white arrow pointing to the large intraperitoneal mass and the red arrow pointing toward the descending/abdominal aorta being shifted laterally off its medial baseline

Due to this concerning finding on abdominal ultrasonography, a CT abdomen and pelvis with contrast was ordered. Imaging demonstrated a large solid and cystic appearing intraperitoneal supra colic mass measuring 15 cm x 22 cm x 23 cm (AP x TV x CC) with cystic necrosis and significant mass effect on the greater curvature of the stomach, porta hepatis, and duodenum (Figures 3-5). Additionally, the radiology report detailed heterogeneous enhancement of the peripheral solid components of the mass. Based on the imaging findings, differential diagnoses included colon cancer, retroperitoneal/peritoneal sarcomas, lymphoma, and other malignancies such as GIST.

During the patient’s hospitalization, interventional radiology performed a biopsy of the abdominal mass. Results from tissue samples were characterized as a spindle cell tumor, suggestive of a GIST. Radiological evidence staged the tumor as III T4N0M0 with next-generation sequencing significant for exon 11 mutation and PDGFRA-mutant negative. The patient deferred treatment options during her hospital stay and was discharged in stable condition after receiving diuretics with the improvement of her lower extremity edema, shortness of breath, and orthopnea.

Upon follow-up with the patient’s primary care provider, the patient endorsed continued improvement of her functional status while on maintenance diuretics. The patient decided against bulk resection of her tumor with the surgical team; however, she started targeted molecular therapy with imatinib 100 mg twice daily. The decision to begin therapy was made in an attempt to reduce the size of the tumor with anticipated resection at a later time.

## Discussion

Since the discovery of GISTs in 1983, medicine’s understanding of this neoplasm has improved significantly. Although a majority of these tumors are benign, the potential for malignant progression is not to be underestimated. Advances in surgical techniques and molecular biology have significantly minimized deleterious outcomes as well as reduced mortality rates of patients diagnosed with GISTs [8]. In many cases, patients are diagnosed incidentally; however, there is a subset of individuals who experience symptoms due to mass effects on neighboring organs. Most of the current literature details that the spectrum of clinical signs associated with GISTs is typically seen in cases where a tumor grows to a size exceeding six centimeters in diameter [8]. Many of the classic symptoms of an intra-abdominal neoplasm of this size, or larger, are like those the case patient presented. Notably, the patient’s abdominal pain, early satiety, distention, and resultant weight loss are considered classic signs. Unfortunately, due to the indolent course of GISTs, many patients may only seek medical attention when tumors have reached a size in which they are able to be palpated or observed externally.

As with other large intra-abdominal lesions, advanced GISTs may result in pernicious outcomes for neighboring organs due to mass effect. In the presented case, the patient had clinical signs of lower extremity edema and endorsed orthopnea as well as dyspnea on exertion. This clinical picture, in conjunction with significant radiologic and laboratory findings, suggested dysfunction of the patient’s cardiopulmonary system. More specifically, the patient's EKG as well as an elevated NT-ProBNP (>1,300 PG/mL) strongly supports myocardial stress and subsequent remodeling. In addition to these findings, radiography performed inpatient demonstrated an enlarged right atrium, enlarged peripheral hypervascularity, and pruning of the pulmonary vessels (Figure 2). Although a formal echocardiogram would be necessary to appropriately diagnose a patient with elevated cardiopulmonary pressures, the presented data and resulting clinical presentation strongly support this proposed conclusion.

There are several theories postulated in the literature that attempt to explain the pathologic cardiopulmonary sequelae resulting from large intra-abdominal tumors. With regard to the presented case, it is suggested that the 23 cm GIST resulted in increased central venous pressure due to IVC and portal vein compression. Literature has detailed the effects of the compression of these vessels as a main contributor to the congestion of the cardiopulmonary systems via several mechanisms. The decreased vessel capacitance of the IVC as well as the portal vein has been suggested to result in compromised function of the splanchnic vasculature and deficient abdominal lymph flow. The resulting congestion contributes significantly to interstitial edema and directly to increased cardiac filling pressures via increased afterload. Moreover, further cardiopulmonary dysfunction was likely the result of increased myocardial afterload due to analogous mechanisms on the abdominal aorta [9,10]. This can be appreciated by the significant deviation of the abdominal aorta from its typical midline position (Figures 4-5).

Another entity that is believed to contribute to cardiovascular compromise in patients with significant abdominal masses is the cephalad deviation of the diaphragm. The mechanism by which this elevates cardiac pressures is well-defined in abdominal compartment syndrome and was initially described over 80 years ago. Along with elevated filling pressures, the diaphragm has also been postulated to cause direct cardiac compression, thus reducing ventricular compliance and contractility [9,10]. The radiology presented in this case demonstrated low lung volumes and an elevated diaphragm suggesting that these mechanisms were likely contributing to the patient's overall clinical picture.

In the presented case, the patient was initially admitted for management of a suspected acute heart failure (HF) exacerbation and was later found to have a 23 cm GIST. Although the primary team believes that the patient’s presenting symptoms of a classic HF exacerbation were precipitated by the GIST, a more comprehensive workup would need to be completed to confirm this suspicion. Despite this limitation, the absence of other comorbid medical conditions in the presented patient makes it difficult to dismiss this probability. Furthermore, the mechanisms proposed are strongly supported by extensive literature detailing the physiology that occurs in abdominal compartment syndrome. This unique case provides insight into the relationship between increased intra-abdominal pressures and disruption of vascular anatomy as a possible cause of acute decompensated HF.

## Conclusions

The complications that arise from large intrabdominal lesions are extensive ranging from minimal symptoms of abdominal pain and distention to emergency situations in cases of hemorrhage. The key aspect of this case that we wish to emphasize is the constellation of symptoms and diagnostic results precipitated by the presented 23 cm GIST. This clinical picture was initially misdiagnosed as a case of new-onset decompensated HF. However, due to a more extensive evaluation, this was found to not be the case. This patient’s clinical course represents the diagnostic challenges of a large GIST and the multidisciplinary approach taken to provide the patient with appropriate information to exercise her autonomy.
